# Actin-binding Rho activating C-terminal like (ABRACL) transcriptionally regulated by MYB proto-oncogene like 2 (MYBL2) promotes the proliferation, invasion, migration and epithelial-mesenchymal transition of breast cancer cells

**DOI:** 10.1080/21655979.2022.2056821

**Published:** 2022-03-28

**Authors:** Jie Li, Hui Chen

**Affiliations:** a Department of Emergency, Hubei Maternal and Child Health Hospital, Tongji Medical College, Huazhong University of Science and Technology, Wuhan, Hubei, China; b College of Medicine, Jiaxing University, Jiaxing, Zhejiang, China

**Keywords:** ABRACL, MYBL2, breast cancer, EMT, gelatin zymography

## Abstract

Breast cancer is the most common malignant tumor in females with high incidence and mortality. Actin-binding Rho activating C-terminal like (ABRACL) was highly expressed in several cancers. We aimed to investigate the function and mechanism of ABRACL in breast cancer. In this study, biological information analysis predicted the expression of ABRACL and MYB proto-oncogene-like 2 (MYBL2) in breast cancer tissues and their possible relationship. With the application of RT-qPCR and western blot, the mRNA and protein expression of ABRACL and MYBL2 in breast cancer cell lines were assessed. After ABRACL interference, an assessment of cell proliferation was carried out using cell counting kit (CCK)-8, colony formation, and western blot. The invasive and migratory abilities of cells were determined by transwell and wound healing assays. The epithelial-mesenchymal transition (EMT) process was assayed utilizing western blot. The relationship between ABRACL and MYBL2 was confirmed by luciferase reporter assay and chromatin immunoprecipitation (ChIP). The above experiments were done again after MYBL2 overexpression in breast cancer cells with ABRACL deletion. Results revealed that ABRACL and MYBL2 were highly expressed in breast cancer tissues and cells. ABRACL knockdown suppressed the proliferation, invasion, migration, and EMT of breast cancer cells. MYBL2 transcriptionally activated ABRACL. Besides, MYBL2 overexpression reversed the effects of ABRACL knockdown on cell malignant biological behaviors. To conclude, ABRACL could be transcriptionally regulated by MYBL2 to promote cell malignant biological behaviors in breast cancer cells, implying the potential of ABRACL being a promising target for the improvement of breast cancer therapy.

## Graphical abstract

**Figure:**
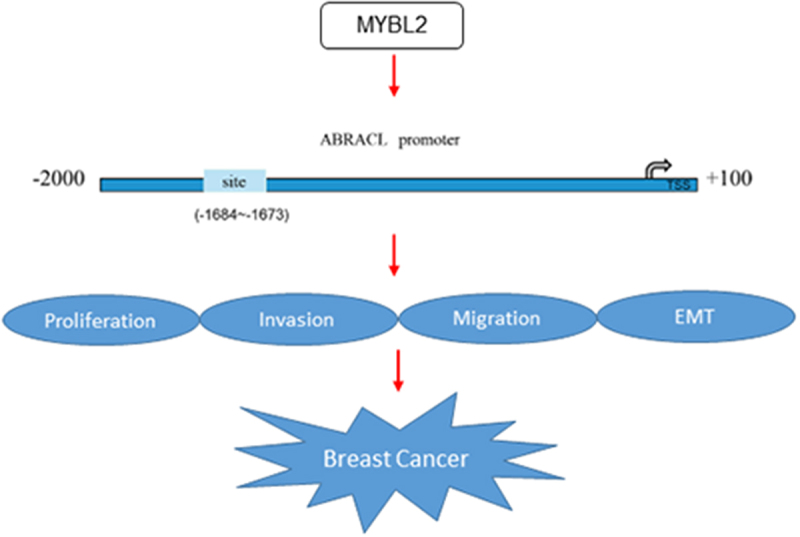

## Introduction

Breast cancer, which poses health risks to women every year, has high prevalence in every corner of the world [1]. Breast cancer is mainly caused by estrogen, and its development is influenced by family history, gender, genetics, and poor lifestyle [2]. Currently, breast cancer treatment is usually based on surgery, targeted therapy, hormone therapy, and early screening [3,4]. However, these treatments cannot prevent the advancement of breast cancer and only works once it has occurred. Only a comprehensive understanding of molecular mechanisms of tumorigenesis, and progressive development of effective approaches, can significantly improve diagnostic and therapeutic outcomes.

It is well known that during the development of cancers, cells undergo proliferation, migration, and invasion to spread into surrounding tissues and blood vessels [5,6]. In addition, epithelial–mesenchymal transition (EMT) plays an important role in the metabolic remodeling of cancer cells as well as in phenotype switching and therapy resistance [7]. Therefore, the suppression of cancer cell proliferation, invasion, migration, and EMT may be an important way to mitigate cancer progression. It has been shown that the regulation of cellular actin dynamics plays a key role in driving cancer cell motility [8]. Thus, dysregulation of actin regulators is associated with cancer development. Actin-binding Rho activating C-terminal-like (ABRACL) is a regulator of the actin cytoskeleton and cell motility and is closely associated with invasion and metastasis of multiple cancers [9]. Several studies found that ABRACL was highly expressed in gastric and colon cancers, and its high expression had close relation with the poor prognosis of patients [9,10]. However, it is not known whether ABRACL affects the progression of breast cancer.

With the purpose of further investigating the molecular mechanism of ABRACL, HumanTFDB (http://bioinfo.life.hust.edu.cn/HumanTFDB#!) database was utilized for prediction, and the results revealed that the transcription factor MYB proto-oncogene-like 2 (MYBL2) might bind to the promoter region of ABRACL. MYBL2, also known as B-MYB, belongs to myelodysplastic transcription factors [11]. A large body of literature has found that MYBL2 overexpression plays an oncogenic role in cancers, such as colorectal cancer and hepatocellular carcinoma [12,13]. MYBL2-induced PITPNA antisense RNA 1 (PITPNA-AS1) upregulates salt-inducible kinase 2 (SIK2) to exert oncogenic functions in triple-negative breast cancer [14]. Besides, MYBL2 was reported to play important regulatory roles in proliferation, differentiation, and cell cycle [15]. Importantly, MYBL2 knockdown imparted suppressive effects on the proliferative ability of breast cancer cells [16]. Based on the above studies, we speculated that ABRACL might be transcriptionally regulated by MYBL2 and promoted the malignant behaviors of breast cancer cells.

In the present study, the expression of ABRACL in breast cancer tissues and overall survival were analyzed by using GEPIA2 database. Then, the functions of ABRACL on malignant biological properties of breast cancer cells were explored. The potential mechanism of ABRACL related to MYBL2 was further studied. Our findings may give rise to a new approach and theoretical basis for the treatment of breast cancer.

## Materials and methods

### Bioinformatics tools

ABRACL and MYBL2 expression in breast cancer tissues and overall survival rate were analyzed by GEPIA2 database (http://gepia.cancer-pku.cn/) [17]. The possible binding sites of transcription factor MYBL2 and ABRACL promoter were predicted by HumanTFDB database (http://bioinfo.life.hust.edu.cn/HumanTFDB#!) [18]. The correlation between ABRACL and MYBL2 expression in breast cancer tissues was analyzed byENCORI database (https://starbase.sysu.edu.cn/index.php) [19].

### Cell culture

Human mammary epithelial cells MCF-10A (cat. No. MCF-10A), human luminal A breast cancer cell line MCF-7 (cat. No. TCHu 74), triple-negative breast cancer cell line MDA-MB-231 (cat. No. TCHu227) and the inflammatory breast cancer cell line SUM190PT (cat. No. CVCL-3423) were all obtained from BioVector NTCC Inc. Dulbecco’s modified Eagle’s medium (DMEM)/F12 (Gibco, Grand Island, NY, USA) mixed medium decorated with 20 ng/mL epidermal growth factor (EGF) was applied to culture MCF-10A cells. MCF-7 and MDA-MB-231 cells were cultured in DMEM that contained 10% heat-inactivated fetal bovine serum (FBS; Gibco, Grand Island, NY, USA). SUM190PT was cultured in Roswell Park Memorial Institute (RPMI) 1640 medium containing 10% FBS. All cell incubation was provided at 37°C in a 95% humidified incubator with 5% CO_2_.

### Cell transfection

MCF-7 cells were plated into 6-well plates (2x10^5^ cells/well) and cultured at 37°C until 80% confluence. Short hairpin RNAs (shRNAs) targeting ABRACL (shRNA-ABRACL-1, 5’-GCTGATGGAAAGTTAAGCGTG-3’; shRNA-ABRACL-2, 5’-GCCAACCTCTTTGAAGCATTG-3’) and its negative control (shRNA-NC), overexpression plasmid of MYBL2 (Ov-MYBL2) and its negative control (Ov-NC) were provided by BioVector NTCC Inc. These designed vectors were subjected to transfection into MCF-7 cells with the help of Lipofectamine 2000 reagent (Invitrogen, Carlsbad, CA, USA) strictly as instructed by the reagent supplier. These transfected MCF-7 cells were available for further experiments after 48 h.

### Reverse transcription-quantitative polymerase chain reaction (RT-qPCR) assay

Total RNAs were extracted from MCF-7 cells employing total RNA Extraction Kit supplied by Solarbio Life Sciences (cat. No. R1200, Beijing, China) following the operating protocols of the vendor. The complementary DNA (cDNA) generation from RNA was carried out with the use of RevertAid First-Strand cDNA Synthesis Kit provided by Fermentas International Inc (cat. No. K1622; Burlington, Ontario, Canada). PCR amplification was executed with the adoption of Power SYBR Green PCR Master Mix (Applied Biosystems, Thermo Fisher Scientific) on an ABI 7300 thermal-recycler (Applied Biosystems; Thermo Fisher Scientific). The amplification process was performed as follows: 40 cycles of 95°C for 15 sec, 60°C for 15 sec and 72°C for 45 sec. The primer sequences were displayed as follows: ABRACL, forward: 5’-ACCTCTTTGAAGCATTGGTAGG-3’, reverse: 5’- GCAGCTCTCCTGGATATGTTAC-3’; MYBL2, forward: 5’-CCGGAGCAGAGGGATAGCA-3’, reverse: 5’-CAGTGCGGTTAGGGAAGTGG-3’; GAPDH, forward: 5’-ACAACTTTGGTATCGTGGAAGG-3’ and reverse: 5’- GCCATCACGCCACAGTTTC-3’. Glyceraldehyde-phosphate dehydrogenase (GAPDH) acted as an endogenous reference. All relative gene expression was subjected to the calculation of 2^–ΔΔCt^ [20].

### Western blot

The proteins that isolated from MCF-7 cells by radioimmunoprecipitation (RIPA) Lysis Buffer (Beyotime, Shanghai, China) were then quantified using bicinchoninic acid (BCA) kit (Thermo Fisher Scientific). After protein sample separation by sodium dodecyl sulfate-polyacrylamide gel electrophoresis (SDS-PAGE), they were transferred onto polyvinylidene fluoride (PVDF) membranes (Merck Millipore, Danvers, MA, US). Samples were blocked using 5% skimmed milk for 1 h before overnight incubation with the primary antibodies against ABRACL (Sigma-Aldrich, cat. No. #HPA030217, 1:2500), MYBL2 (Abcam, ab12296, 1:2500), Ki67 (Abcam, ab92742, 1:5000), proliferating cell nuclear antigen (PCNA; Abcam, ab92552, 1:1000), matrix metallopeptidase 2 (MMP2; Abcam, ab92536, 1:1000), MMP9 (Abcam, ab76003, 1:1000), E-cadherin (Abcam, ab133597, 1:1000), Snail (Abcam, ab216347, 1:1000), Vimentin (Abcam, ab92547, 1:1000), N-cadherin (Abcam, ab76011, 1:5000) at 4°C. Prior to the rinse with phosphate buffer solution (PBS), HRP-labeled goat anti-rabbit secondary antibody (Beyotime, cat. No. A0208, 1:1000) was applied to foster the membranes at room temperature for 2 h. The protein blots were detected with the application of an enhanced chemiluminescent (ECL) Chemiluminescence Detection Kit (PromoCell, cat. No. PK-MB902-500-500) and subjected to analysis by Image Lab Software (Bio-Rad, Hercules, CA, US).

### Cell counting kit (CCK)-8

MCF-7 cells that inoculated into 96-well plates (5x10^3^ cells/well) were incubated for 24, 48, and 72 h at 37°C with 5% CO_2_. 10 μL of CCK-8 reagent (Yeasen Biotechnology Co., cat. No. 40203ES60, Shanghai, China) was added in each well for incubation together with the cells for 4 h. Finally, the absorbance value was determined with the help of a microplate reader (Bio-Rad, Hercules, CA, USA).

### Colony formation assay

The incubation of MCF-7 cells (1x10^3^ cells/well) which were inoculated into 6-well plates lasted 14 days. Subsequently, PBS was adopted to wash the cells, after which were the fixation and staining with 4% formaldehyde and 0.5% crystal violet, individually. The number of colonies formed more than 50 cells/colony was counted with an Olympus BX40 light microscope (Olympus, Tokyo, Japan).

### Wound healing assay

Transfected MCF-7 cells (1x10^3^/well) were seeded in 6-well plates at 37°C with 5% CO_2_. When the cell confluence exceeded 95%, a wound healing assay was carried out by scratching the cell monolayer with a sterile 200-μL gun tip. Cells were washed with D-hanks and then incubated by adding low concentrations of serum. Photographs of cell migration were taken at 0 and 24 h under a light microscope (Olympus, Tokyo, Japan) and statistical analysis was performed using Image J software (National Institutes of Health).

### Transwell assay

MCF-7 cells (5x10^4^ cells per well) suspended in 200 μL of serum-free medium were added to the upper layer of transwell chambers covered with 10% Matrigel (BD, Franklin Lakes, NJ, USA). The lower layer was then added with 600 µL of medium containing 20% FBS. When the cells were filtered to the bottom of the chambers, 4% paraformaldehyde and 0.5% crystal violet were applied to fix and stain the cells separately. Five random areas of each group were photographed under a light microscope (Olympus, Tokyo, Japan), and the number of cells was counted.

### Luciferase reporter assay

Luciferase reporter plasmids (Promega Corporation) were constructed with wild-type (WT) and mutant-type (MUT) regions of ABRACL promoter. MCF-7 cells (1 × 10^3^cells/90 μL/well) in each group were cultured overnight in 96-well plates until growing to 70% confluence. Cells were subsequently transfected with Ov-MYBL2 or Ov-NC and WT or MUT ABRACL promoter regions using Lipofectamine® 2000 reagent (Invitrogen; Thermo Fisher Scientific, Inc.). After 48 h, the Dual-Luciferase reporter assay system (Promega Corporation) was employed to estimate the luciferase activity.

### Chromatin immunoprecipitation (ChIP)

MYBL2 and ABRACL-binding promoters were assayed by ChIP. Briefly, MCF-7 cells got fixed with 1% formaldehyde. After termination of cross-linking, DNA was sheared into 200 bp to 500 bp fragments using VCX750 ultrasound on ice. After performing centrifugation, NaCl at a final concentration of 0.2 M was added and un-crosslinked at 65°C for 2 h. Thereafter, 60 uL ProteinA Agarose/SalmonSperm DNA was added to each tube and shaken gently at 4°C for 2 h. The immunoprecipitation of the lysate was performed with MYBL2 antibody. After elution, the lysate was un-crosslinked with 20 µl of NaCl overnight at 65°C. Subsequently, each tube was decorated with 1 µL RNaseA (MBI) for incubation. DNA was recovered for PCR reaction.

### Statistical analysis

The analysis of all experimental data relied on the GraphPad Prism 8.0 software (GraphPad Software, San Diego, CA, USA). The data were conformed to normal distribution using Shapiro–Wilk test and were presented in the form of mean ± standard deviation (SD). Comparisons between the two groups were made using Student’s t-test, and comparisons among multiple groups were made using one-way analysis of variance (ANOVA) followed by Tukey’s post hoc test. The value of P < 0.05 for each group were judged to be of statistical significance.

## Results

### ABRACL is highly expressed in breast cancer tissues and cell lines

**Figure 1.**
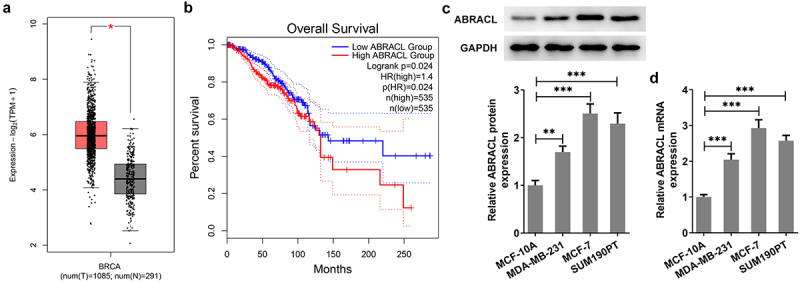
ABRACL is highly expressed in breast cancer tissues and cell lines. (a-b) ABRACL expression in breast cancer tissues and the overall survival as showed in GEPIA2. (c-d) ABRACL mRNA and protein expression in breast cancer cell lines was examined with the use of western blot and RT-qPCR. **P < 0.01, ***P < 0.001.

It has been reported that LEMD1 ABRACL is a regulator of the actin cytoskeleton and cell motility and is closely associated with invasion and metastasis of human cancers [9]. It was observed that ABRACL was highly expressed in breast cancer tissues by GEPIA2 database (http://gepia.cancer-pku.cn/) (Figure 1a). The high expression of ABRACL contributed to a significantly decreased overall survival of breast cancer patients (Logrank P = 0.024; Figure 1b). To figure out ABRACL expression in breast cancer cell lines, we examined ABRACL expression in normal breast epithelial cell line MCF-10A and several representative cell lines MDA-MA-231, MCF-7, and SUM190PT one by one. From Figure 1c-d, it could be clearly seen that ABRACL expression was notably upregulated in these breast cancer cell lines compared with the MCF-10A group. Importantly, the highest ABRACL expression was observed in MCF-7 cells. Therefore, MCF-7 cells were selected for subsequent experiments. Based on the above studies, it can be concluded that ABRACL has abundant expression in breast cancer cells.

### ABRACL knockdown inhibits the level of proliferation in breast cancer cells

**Figure 2.**
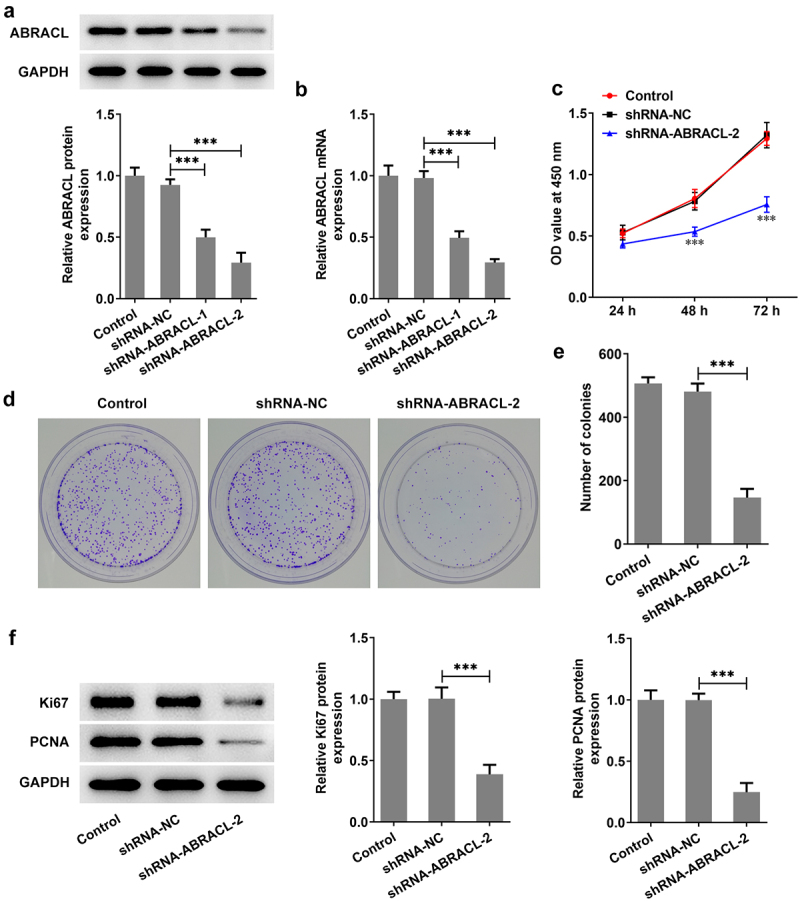
ABRACL knockdown inhibits the level of proliferation in breast cancer cells. (a-b) ABRACL protein and mRNA expression in MCF-7 cells after ABRACL knockdown was examined with the use of western blot and RT-qPCR. ***P < 0.001. (c) The proliferation of MCF-7 cells after ABRACL knockdown was assessed by CCK-8. ***P < 0.001 vs. shRNA-NC. (d-e) The proliferation of MCF-7 cells after ABRACL knockdown was tested by the way of colony formation assay. (f) The expression of Ki67 and PCNA was detected with the adoption of western blot. ***P < 0.001.

For the sake of figuring out whether ABRACL had an effect on the proliferative ability of breast cancer cells, we first knocked down ABRACL expression in MCF-7 cells. It was found that MCF-7 cells transfected with sh-ABRACL-2 exhibited lower ANRACL expression than those transfected with sh-ABRACL-1 (vs sh-NC; Figure 2a-b). Therefore, sh-ABRACL-2 with better transfection efficacy was utilized for subsequent experiments. Subsequently, CCK-8 was adopted for the detection of the proliferation of MCF-7 cells at 24, 48, and 72 h. Obviously, the proliferation level of MCF-7 cells transfected with sh-ABRACL-2 was gradually decreased in a time-dependent manner compared with the sh-NC group as seen in Figure 2c. Similarly, the sh-ABRACL-2 transfection resulted in a lower number of colonies in MCF-7 cells compared with the sh-NC group (Figure 2d-e). Additionally, the levels of proliferation-related proteins including Ki67 and PCNA were also markedly decreased in the shRNA-ABRACL-2 group in comparison with those in the sh-NC group (Figure 2f). Overall, these findings highlight the inhibitory effects of ABRACL knockdown on breast cancer cell proliferation.

### Knockdown of ABRACL inhibits invasion, migration, and EMT of breast cancer cells

**Figure 3.**
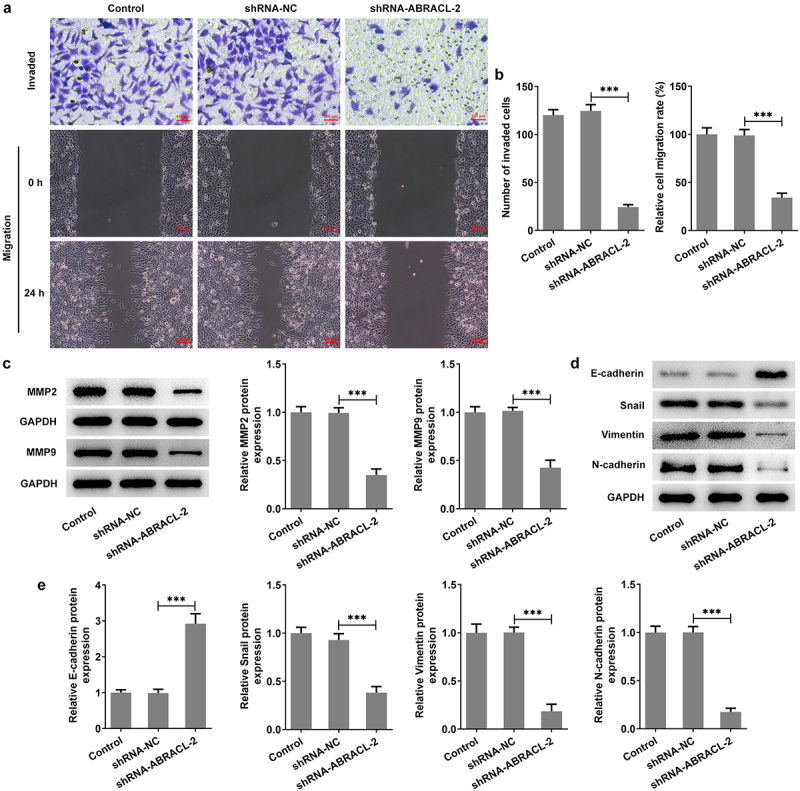
Knockdown of ABRACL inhibits the invasion, migration and EMT of breast cancer cells. (a) The invasive ability of MCF-7 cells after ABRACL knockdown was assayed by transwell experiment. (b) Cell migration capacity of MCF-7 cells after ABRACL knockdown was determined by wound healing experiment. (c) The expression of MMP2 and MMP9 in MCF-7 cells after ABRACL knockdown was examined by means of western blot. (d-e) The expression of E-cadherin, Snail, Vimentin and N-cadherin in MCF-7 cells after ABRACL knockdown was tested employing western blot. ***P < 0.001.

This section continued to estimate the impacts of ABRACL silencing on the biological behaviors of breast cancer cells. It was clearly observed in Figure 3a-b that both cell invasion and migration were reduced after knocking down the expression of ABRACL compared to the sh-NC group. Correspondingly, the levels of migration-associated proteins MMP2 and MMP9 were also decreased in contrast with the sh-NC group (Figure 3c). Furthermore, by examining the contents of EMT-related proteins, we found that ABRACL knockdown led to a rapid increase in E-cadherin expression and a dramatic decrease in the expression of Snail, Vimentin, and N-cadherin in contrast with that in sh-NC group (Figure 3d-e). Obviously, ABRACL knockdown appears to inhibit the relative invasion and migration rate as well as EMT in breast cancer cells.

### MYBL2 can transcriptionally activate ABRACL expression

**Figure 4.**
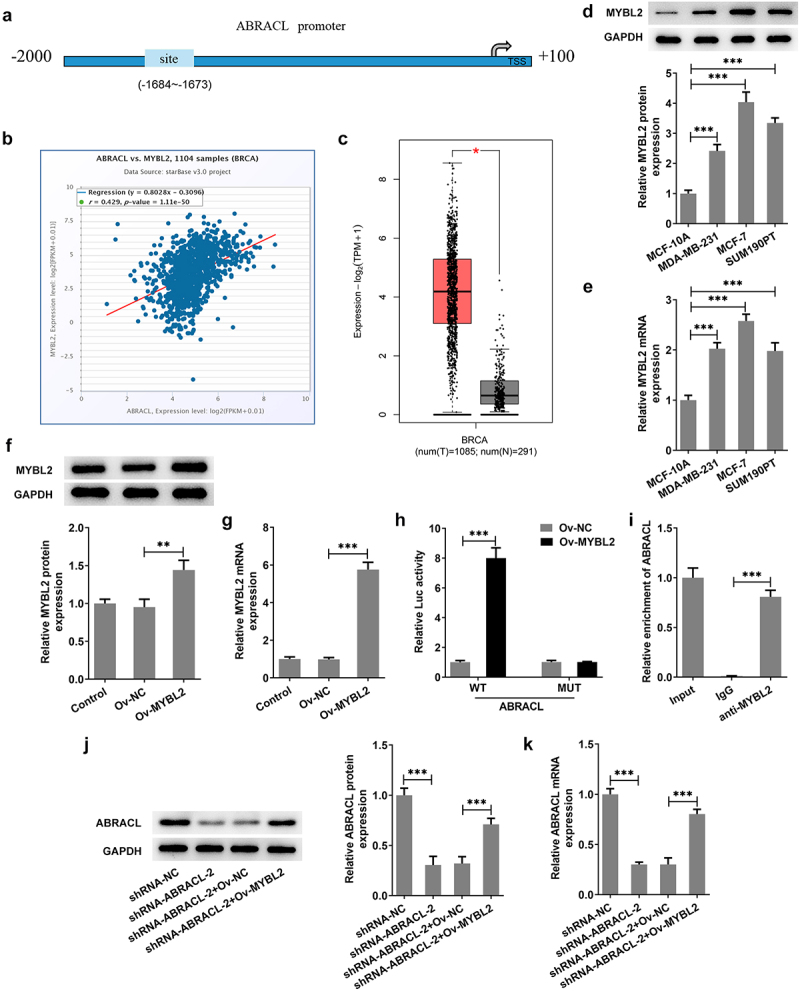
MYBL2 can transcriptionally activate ABRACL expression. (a) Binding sites of MYBL2 and ABRACL promoters were predicted by HumanTFDB website. (b) The relationship of MYBL2 and ABRACL was determined with the help of ENCORI database. (c) MYBL2 expression in breast cancer tissues was analyzed in GEPIA2 database. (d-e) MYBL2 protein and mRNA expression in breast cancer cell lines was examined with the employment of western blot and RT-qPCR. (f-g) MYBL2 expression in transfected breast cancer cells was examined with the employment of western blot and RT-qPCR. (h) The relative promoter activity was detected by luciferase reporter gene assay. (i) The relative enrichment intensity of ABRACL was determined by ChIP. (j-k) ABRACL expression in MCF-7 cells transfected with shRNA-ABRACL-2 and Ov-MYBL2 was subjected to detection with the application of western blot and RT-qPCR. **P < 0.01, ***P < 0.001.

To explore the potential mechanisms of ABRACL on the regulation of breast cancer progression, the human TFDB database was employed to predict the transcription factors that could regulate ABRACL expression. We found that MYBL2 could bind to the promoter of ABRACL (Figure 4a). Meanwhile, results on ENCORI website showed that MYBL2 expression was positively correlated with ABRACL expression (r = 0.429, P = 1.11e-50; Figure 4b). Subsequently, GEPIA2 database revealed that MYBL2 expression gained a huge growth in breast cancer tissues as well (Figure 4c). Further validation implied that MYBL2 expression was indeed much higher in MCF-7 cells than in other three cells (Figure 4d-e). Given that the expression of ABRACL and MYBL2 was upregulated in breast cancer cells, the following experiments were conducted to verify whether there was a link between them. The overexpression plasmid of MYBL2 was transfected into MCF-7 cells, and notably elevated MYBL2 expression was observed in the Ov-MYBL2 group compared with the Ov-NC group (Figure 4f-G). Then, it was noticed that MYBL2 overexpression significantly increased the relative fluorescence activity in ABRACL-WT group but exerted no significant influence on ABRACL-MUT group (Figure 4h). In addition, the relative enrichment of ABRACL was elevated in the anti-MYBL2 group relative to the IgG group (Figure 4i). Apart from this, MYBL2 overexpression elevated the protein and mRNA expression of ABRACL in MCF-7 cells transfected with shRNA-ABRACL-2 (Figure 4j-k). Collectively, the above-mentioned findings outline that MYBL2, a transcription factor, activates ABRACL expression in breast cancer cells.

### Overexpression of MYBL2 reverses the effect of ABRACL knockdown on the proliferation of breast cancer cells

**Figure 5.**
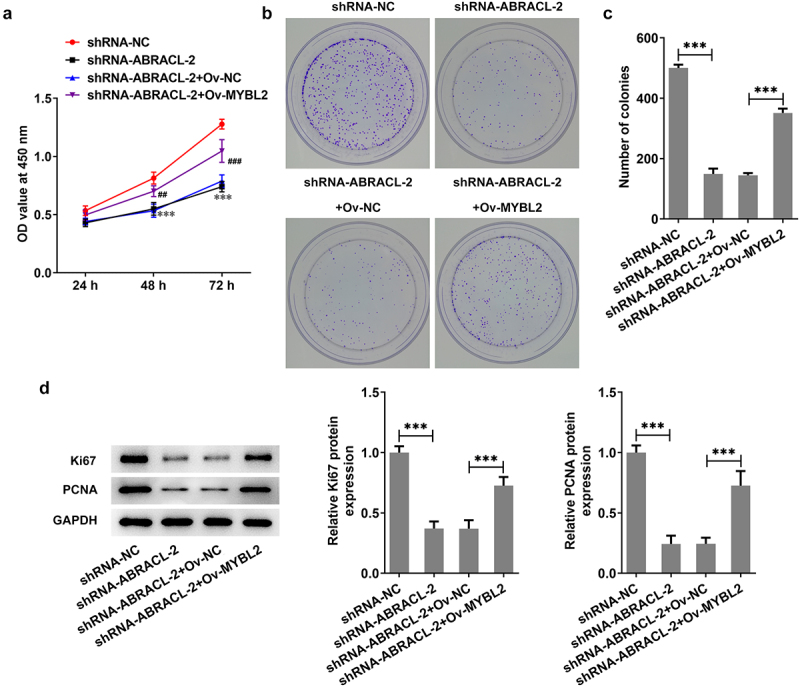
Overexpression of MYBL2 reverses the effect of ABRACL knockdown on the proliferation of breast cancer cells. (a) The proliferation of MCF-7 cells after transfection with shRNA-ABRACL-2 and Ov-MYBL2 was assessed by CCK-8. ***P < 0.001 vs. shRNA-NC; ##P < 0.01, ###P < 0.001 vs. shRNA-ABRACL-2+ Ov-NC. (b-c) The ability of colony formation in MCF-7 cells transfected with shRNA-ABRACL-2 and Ov-MYBL2 was tested by the way of colony formation assay. (d) The expression of Ki67 and PCNA in MCF-7 cells transfected with shRNA-ABRACL-2 and Ov-MYBL2 was detected with the adoption of western blot. ***P < 0.001.

Previous findings confirmed that ABRACL knockdown could inhibit the malignant behaviors and EMT of breast cancer cells and was transcriptionally regulated by MYBL2. Here, we investigated whether MYBL2 overexpression could stimulate the proliferative ability of breast cancer cells. It was apparently observed in Figure 5a that MCF-7 cells co-transfected with shRNA-ABRACL-2 and Ov-MYBL2 showed a higher level of cell proliferation than those transfected with shRNA-ABRACL-2 and Ov-NC. Similarly, the ability of colony formation was higher in shRNA-ABRACL-2+ Ov-MYBL2 group in comparison with that in shRNA-ABRACL-2+ Ov-NC group (Figure 5b-c). What is more, the levels of proliferation-related proteins Ki67 and PCNA were also elevated after transfection with shRNA-ABRACL-2 and Ov-MYBL2 (Figure 5d). All findings suggest that overexpression of MYBL2 partially reverses the regulatory effects of ABRACL knockdown on the proliferation of breast cancer cells.

### Overexpression of MYBL2 reverses the effects of ABRACL knockdown on the invasion, migration, and EMT of breast cancer cells

**Figure 6.**
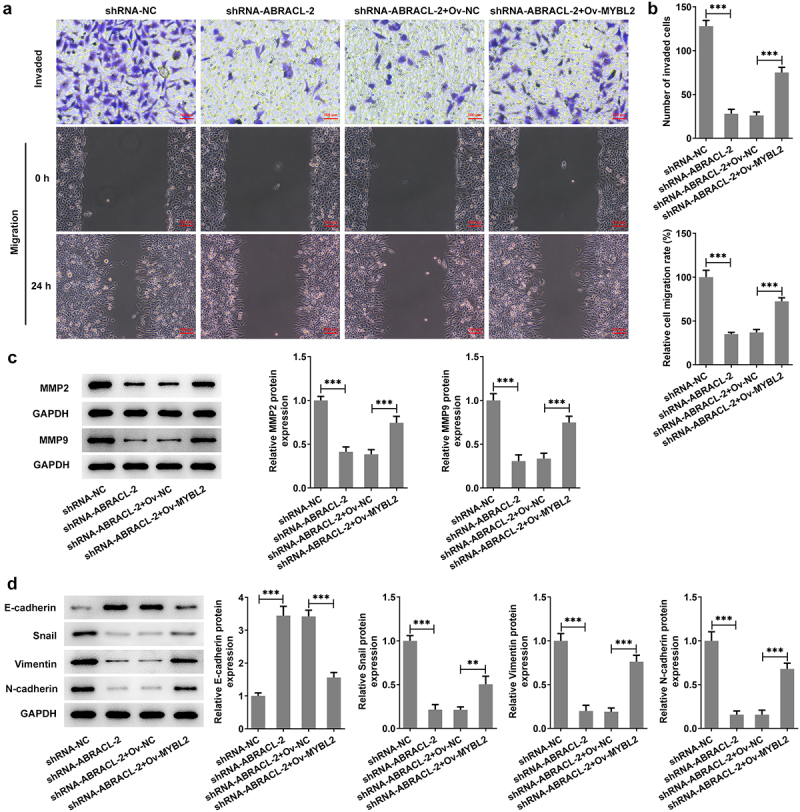
Overexpression of MYBL2 reverses the effects of ABRACL knockdown on the invasion, migration and EMT of breast cancer cells. (a) The invasive ability of MCF-7 cells transfected with shRNA-ABRACL-2 and Ov-MYBL2 was assayed by transwell experiment. (b) The migratory capacity of MCF-7 cells transfected with shRNA-ABRACL-2 and Ov-MYBL2 was determined by wound healing experiment. (c) The expression of MMP2 and MMP9 in MCF-7 cells transfected with shRNA-ABRACL-2 and Ov-MYBL2 was examined by means of western blot. (d) The expression of E-cadherin, Snail, Vimentin and N-cadherin in MCF-7 cells transfected with shRNA-ABRACL-2 and Ov-MYBL2 was tested employing western blot. **P < 0.01, ***P < 0.001.

This section investigates the possible effects of MYBL2 overexpression on the invasion, migration, and EMT process of ABRACL-knockdown MCF-7 cells. Significant increase in the levels of cell invasion and migration was found in shRNA-ABRACL-2+ Ov-MYBL2 group, in contrast with that in shRNA-ABRACL-2+ Ov-NC group (Figure 6a-b). In addition, the expression of MMP2 and MMP9 was also relatively elevated in MCF-7 cells after transfection with shRNA-ABRACL-2 and Ov-MYBL2, in comparison with the shRNA-ABRACL-2 and Ov-NC group (Figure 6c). More importantly, the EMT marker E-cadherin level was reduced but the levels of Snail, Vimentin, and N-cadherin were improved in MCF-7 cells co-transfected with shRNA-ABRACL-2 and Ov-MYBL2 (Figure 6d). The evidences showed in this section note that MYBL2 overexpression reverses the regulatory effects of ABRACL knockdown on the relative invasion and migration rate, as well as EMT process of breast cancer cells.

## Discussion

Breast cancer is a common gynecological malignancy and a serious threat to women’s health worldwide [21]. The malignant biological behaviors of cancer cells such as proliferation, invasion, and migration and EMT are integral and important parts of cancer development process [22,23]. Therefore, finding effective molecular targets to inhibit the malignant biological behaviors and EMT process of breast cancer cells is very necessary.

Previously, it has been reported that ABRACL was highly expressed in a variety of cancers and was associated with poor patient prognosis [9,10]. We confirmed that ABRACL was also highly expressed in breast cancer tissues by checking the GEPIA database. The ensuing *in vitro* experiments also demonstrated the high content of ABRACL in breast cancer cells. ABRACL is a conserved regulator of actin and cell migration [9]. It is well documented that actin provides mechanical support and motor drive for cells by forming filaments [24]. Additionally, actin and the microtubule cytoskeleton are essential for the proliferative ability, relative invasiveness, and migration rate of cancer cells [25,26]. This evidence suggests that, as a regulator of actin, ABRACL may also be involved in regulating the malignant biological behaviors of cancer cells. Recent literature has revealed that cancer cells with high ABRACL expression have a greater ability to migrate and proliferate [9]. The effects of miR-145-5p interference on cell proliferation, migration, and invasion could be attenuated after ABRACL knockdown [27]. Thus, our study also evidenced that ABRACL silence repressed the proliferative ability, relative migration rate, as well as invasion of breast cancer cells.

EMT is featured by more non-motile epithelial cells than motile mesenchymal cells and contributes pathologically to the development of fibrosis and cancers [28,29]. Cancer cells that have undergone EMT are more aggressive [30]. Therefore, the inhibition of the EMT process is also an important way to block the metastasis of breast cancer cells. It has been claimed that ABRACL can interact with Cofilin, which promotes invasion and migration in response to EMT-inducing factor TGF-β in the microenvironment [9,31,32]. The above clues imply that ABRACL may also be involved in the induction of the EMT process. Additionally, the EMT is characterized by the loss of E-cadherin as well as the gain of Vimentin and N-cadherin [33]. Meanwhile, the EMT process is accompanied by the activation of Snail [34]. In our experiments, the enhanced E-cadherin expression as well as the reduced expression of Snail, Vimentin, and N-cadherin also suggested that knockdown of ABRACL inhibited the EMT process in breast cancer cells, which was in line with the above study.

According to the data on HumanTFDB website, MYBL2 was found to bind to the promoter of ABRACL, and ENCORI database showed a positive correlation between these two genes. This implied that MYBL2 expression was increased with elevated ABRACL expression. An increasing number of research studies have validated that MYBL2 overexpression plays an oncogenic role in cancers like triple negative breast cancer, colorectal cancer, and hepatocellular carcinoma [12–14]. Our experiments disclosed that MYBL2 could be widely found in breast cancer cells. Subsequently, overexpression of MYBL2 increased ABRACL promoter activity. Furthermore, MYBL2 overexpression also upregulated the content of ABRACL, suggesting that the high expression of ABRACL might contribute to the malignant biological behaviors and EMT process of breast cancer cells as well. Furthermore, MYBL2 stimulates the transcription of target genes and promotes the entry of breast cancer into the S and M phases of the cell cycle, cell proliferation, migration, and invasion [35]. Not only that, MYBL2 interacts with STRAP in the cytoplasm and regulates STRAP-mediated TGF-β signaling, thereby inducing EMT process [36]. Our experiments revealed that overexpression of MYBL2 did partially restore the malignant biological behaviors as well as the EMT process in breast cancer cells after ABRACL knockdown.

## Conclusion

In view of all that has been discussed so far, we can conclude that ABRACL can be transcriptionally regulated by MYBL2 to promote the proliferative ability, relative migration rate, invasive ability, as well as EMT process of breast cancer cells. Meanwhile, this study highlights that ABRACL acts as a novel molecular player in breast cancer pathogenesis.

## Data Availability

The data presented in this study are available on request from the corresponding author.
